# Supplementary material for: Why did informal sector workers stop paying for health insurance in Indonesia? Exploring enrollees’ ability and willingness to pay
Source: PLoS One. 2021 Jun 4;16(6):e0252708. doi: 10.1371/journal.pone.0252708 (PMC8177660; doi:10.1371/journal.pone.0252708)
Supplement: S1 Appendix — (DOCX) [file pone.0252708.s001.docx]

# S1 Appendix Calculation of fiscal capacity and fiscal capacity index:

$$KF= \frac{\left( PAD+DAU+DBH+Otsus+Transfer Prov+LP \right)-BP}{Number of poor people}$$

KF = Fiscal capacity
PAD = Local revenue
DAU = General allocation fund
DBH = Profit sharing fund
Otsus = Special autonomy fund
Transfer Prov = Transfer from provincial government to district/city
LP = Other legal revenue
BP = Employee expenditures

$$IKF= \frac{KF}{\left( \Sigma KF \right)/n}$$

IKF = Fiscal capacity index
n = Total number of districts/cities
