# Supplementary material for: Why did informal sector workers stop paying for health insurance in Indonesia? Exploring enrollees’ ability and willingness to pay
Source: PLoS One. 2021 Jun 4;16(6):e0252708. doi: 10.1371/journal.pone.0252708 (PMC8177660; doi:10.1371/journal.pone.0252708)
Supplement: S1 File — (PDF) [file pone.0252708.s002.pdf]

Pusat KP-MAK
PUSAT KEBIJAKAN PEMBIAYAAN DAN MANAJEMEN ASURANSI KESEHATAN
FAKULTAS KEDOKTERAN UNIVERSITAS GADJAH MADA

BPJS Kesehatan
Badan Penyelenggara Jaminan Sosial

QUANTITATIVE INSTRUMENT

Research on Underprivileged Non-Recipient Worker (PBPU) Participants' Ability and Willingness to Pay JKN Premium

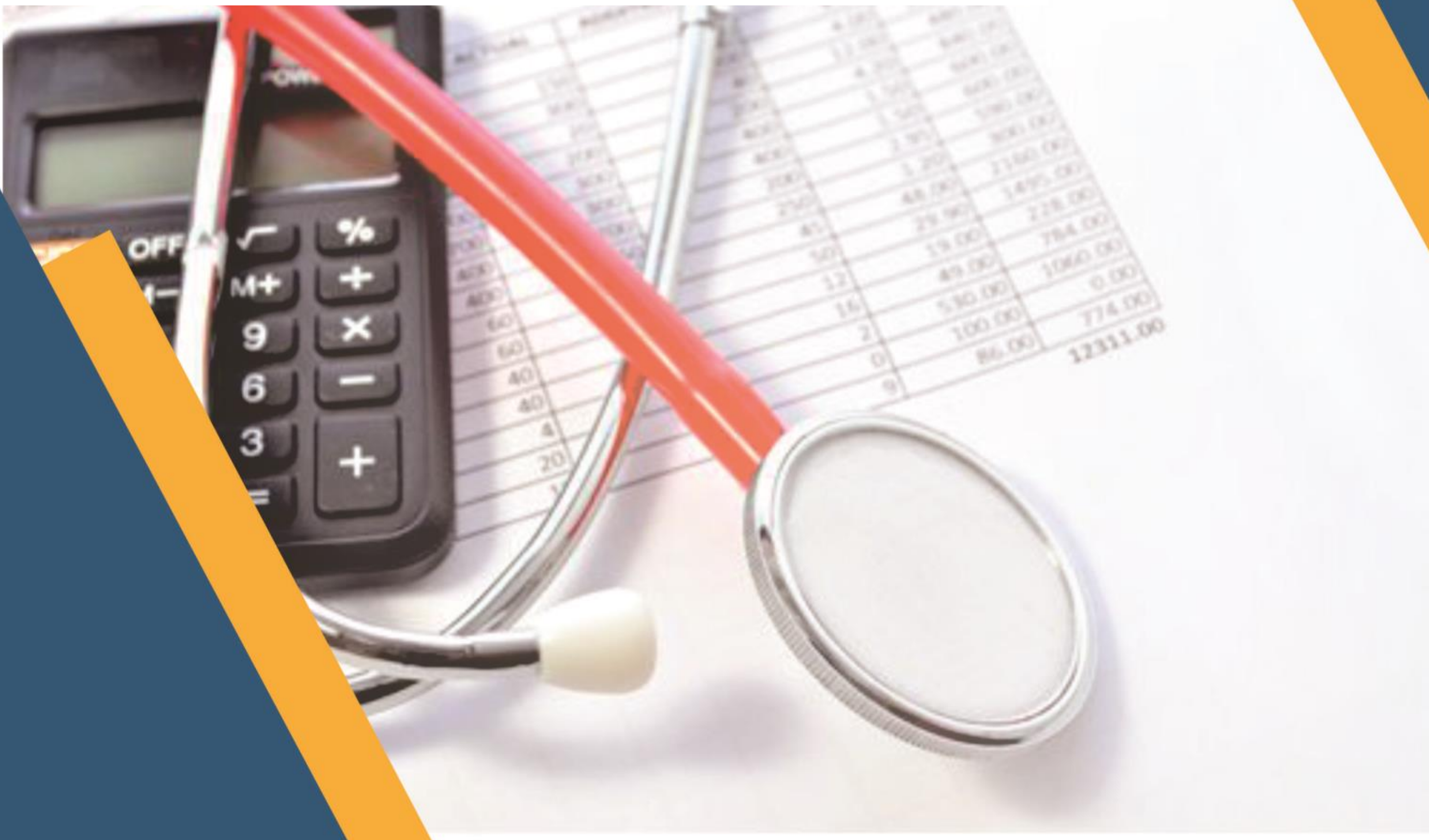

0274 - 631022
pusatkpmak@ugm.ac.id

Faculty of Medicine, Universitas Gadjah Mada
Radioputro Building, 2nd floor, West Wing
Farmako street, Sekip Utara, Special Region of Yogyakarta, Indonesia, 55281

HOUSEHOLD HEALTH SURVEY

Center for Health Financing Policy and Health Insurance Management

Faculty of Medicine-Universitas Gadjah Mada

(Pusat KPMAK FK UGM)

We, Pusat KPMAK FK UGM, are conducting a household health survey. Thus, we ask you to take part as a participant of this research. Please provide relevant information for each item.

Your participation in this study is voluntary. It is up to you to decide whether or not to take part in this study. Withdrawing from this study will not affect the relationship you have, if any, with the researcher. We convince you that every provided information will become valuable inputs for the decision-maker of The Republic of Indonesia. Your responses to this survey will be anonymous. Every effort will be made by the researcher to preserve your confidentiality. Participant data will be kept confidential by assigning unique identity codes.

By signing this form, you have read and understood the provided information, and have had the voluntary opportunity to participate in this survey. After signing the consent form, you are still free to withdraw at any time and without giving a reason.

Participant Name ______________

Participant Signature ______________

Date ______________

Thank you for your cooperation.

Interviewer's Signature

Name ______________

HOUSEHOLD HEALTH SURVEY

PUSAT KP-MAK FK UGM

Questionnaire		
PARTICIPANT NUMBER [ ][ ][ ]	DATE:	SURVEYOR NAME:

I. KETERANGAN TEMPAT

KT01 Province		CODE: [ ][ ][ ]
KT02 Regency/Municipality		CODE: [ ][ ][ ]
KT03 Sub-district		
KT04 Rural/Urban Village		
KT05 Neighborhood (RW)		
KT06 Phone Number		

Code KT01	Code KT02		
12.North Sumatera	1212. Deli Serdang	5103. Badung	7102. Minahasa
14.Riau	1213. Langkat	5104. Gianyar	7106. North Minahasa
35.East Java	1216. Pakpak Bharat	5108. Buleleng	7171. Manado City
36. Banten	1401. Kuantan Singingi	5312. Ngada	7205. Donggala
51. Bali	1405. Siak	5314. Rote Ndao	7208. Tojo Una Una
53. East Nusa Tenggara	1473. Dumai City	5371. Kupang City	7271. Palu City
61. West Kalimantan	3529. Sumenep	6101. Sambas	9419. Sami
64. East Kalimantan	3576. Mojokerto City	6112. Kubu Raya	9420. Kerom
71. North Sulawesi	3578. Surabaya City	6172. Singkawang City	9471. Jayapura City
72. Central Sulawesi	3601. Pandeglang	6405. Berau	9105. Manokwari City
91. West Papua	3604. Serang	6409. North Penajam Paser	9108. Raja Ampat
94. Papua	3674. South Tangerang City	6472. Samarinda City	9171. Sorong City

II. PARTICIPANT IDENTITY

IR01 Participant Age		
IR02 Gender		CODE: [ ][ ][ ]
IR03 Latest Education		CODE: [ ][ ][ ]
IR04 Head of Family's Job		CODE: [ ][ ][ ]
IR05 Wife's Job		CODE: [ ][ ][ ]
IR06 Family income average per month<br>a. Husband (IDR)<br>b. Wife (IDR)	[ ][ ].[ ][ ][ ].[ ][ ][ ]<br>[ ][ ].[ ][ ][ ].[ ][ ][ ]<br>[ ][ ].[ ][ ][ ].[ ][ ][ ]	

c. Other family member (IDR)		
IR07 Number of family member		
IR08 School-age children<br><br>a. 1st child<br><br>b. 2nd child<br><br>c. 3rd child<br><br>d. 4th child	0. No 1. Yes<br><br>Level:<br>Grade:<br><br>Level:<br>Grade:<br><br>Level:<br>Grade:<br><br>Level:<br>Grade:	CODE: ☐☐☐
IR09 Home Ownership Status	0. Rental<br>1. Private-owned<br>2. Office-owned<br>3. Government-owned<br>4. Others, mention	CODE: ☐☐☐
IR10 How many family members that have owned Health JKN/BPJS card?	0. One<br>1. Two<br>2. Three<br>3. Four<br>4. Five<br>5. Six<br>6. More than six	CODE: ☐☐☐

**Code IR02**<br>0. Male<br>1. Female<br><br>**Code IR03**<br>0. Ungraduated from Elementary School<br>1. Elementary School<br>2. Junior High School<br>3. Senior High School<br>4. D1/D2/D3<br>5. Bachelor Degree (S1)	6. Master Degree (S2)<br>7. Doctoral Degree (S3)<br><br>**Code IR04**<br>0. Street vendor<br>1. Laborer<br>2. Retiree<br>3. Farmer<br>4. Fisherman<br>5. Workman<br>6. Temporary worker	7. Private employee<br>8. Entrepreneur / Seller<br>9. Housewife<br>10. Student<br>11. Spiritual leader<br>12. Unemployed<br>13. Others<br><br>**Code IR05**<br>0. Street vendor<br>1. Laborer	2. Retiree<br>3. Farmer<br>4. Fisherman<br>5. Workman<br>6. Temporary worker<br>7. Private employee<br>8. Entrepreneur / Seller<br>9. Housewife<br>10. Student<br>11. Spiritual teacher<br>12. Unemployed<br>13. Others

III. ESTIMATED COST OF HEALTHCARE

Within one month, how many times, on average, the family members receive outpatient care?		
TK01 Puskesmas (Community Health Center)	0. Never<br>1. Once<br>2. Twice<br>3. Three times<br>4. More than three times	CODE: ☐☐☐
TK02 Clinic	0. Never<br>1. Once<br>2. Twice<br>3. Three times<br>4. More than three times	CODE: ☐☐☐
TK03 Hospital	5. Never	CODE: ☐☐☐

	6. Once<br>7. Twice<br>8. Three times<br>9. More than three times	
Within one year, how many times, on average, do the family members receive inpatient care?		
TK04 Community Health Center (Puskesmas)	0. Never<br>1. Once<br>2. Twice<br>3. Three times<br>4. More than three times	CODE: ☐☐☐
TK05 Clinic	0. Never<br>1. Once<br>2. Twice<br>3. Three times<br>4. More than three times	CODE: ☐☐☐
TK06 Hospital	0. Never<br>1. Once<br>2. Twice<br>3. Three times<br>4. More than three times	CODE: ☐☐☐
Within one past month, how many times, on average, did the family members take medication outside the health facility?		
TK07 Buy medicine at a drug store	0. Never<br>1. Once<br>2. Twice<br>3. Three times<br>4. More than three times	CODE: ☐☐☐
TK08 Alternative medicine	0. Never<br>1. Once<br>2. Twice<br>3. Three times<br>4. More than three times	CODE: ☐☐☐

IV. RISK MANAGEMENT

PR01 Based on your experience, how did you pay the healthcare cost when a family member get sick?	0. own money<br>1. helped by relatives<br>2. helped by neighbors<br>3. helped by office/organization<br>4. helped by government<br>5. paid by health insurance<br>6. others, mention	CODE: ☐☐☐
PR02 In terms of cost, how would you feel when a family member gets sick and needs to be hospitalized?	0. I am not worried at all<br>1. I am not worried<br>2. I am quite worried<br>3. I am very worried	CODE: ☐☐☐
PR03 To anticipate that, what would yo do?	0. Save for health cost<br>1. Sell things<br>2. borrow money<br>3. expect help from others<br>4. join health insurance<br>5. No plans<br>6. others, mention	CODE: ☐☐☐

V. INSURANCE

A01. Do you have Healthcare and Social Security Agency (BPJS Kesehatan) card?	0. No<br>1. Yes	CODE: ☐☐☐
A02. If yes, in which class?<br>(If not, skip to question A17)	0. Class I<br>1. Class II<br>2. Class III	CODE: ☐☐☐
A03. If you choose class III, what is your reason?<br>(Only for those answering A02 item: Class III)	0. Most affordable premium<br>1. Shorter waiting time<br>2. Follow my neighbors/friends<br>3. Others, mention	CODE: ☐☐☐
A04. What is your reason becoming a JKN/BPJS participant?	0. Government program/ required by the government<br>1. A family member needs medication<br>2. Anticipate future needs of medication<br>3. Follow my neighbors/friend<br>4. Others, mention	CODE: ☐☐☐
A05. How did you get information about JKN/BPJS?	0. Explanation from BPJS<br>1. Government Unit<br>2. Mass media<br>3. Friends/neighbors<br>4. Others, mention	CODE: ☐☐☐
A06. In what year did you start registering as a JKN/BPJS participant?	0. 2014<br>1. 2015<br>2. 2016	CODE: ☐☐☐
A07. Do you pay Health JKN/BPJS premium regularly?	0. No<br>1. Yes	KODE: ☐☐☐
A08. If yes, what is the reason?<br>(Skip to item A16)	0. It is an obligation<br>1. To prevent penalties<br>2. To easily access healthcare<br>3. Others, mention	CODE: ☐☐☐
A09. If not, what is the reason?<br>(You can provide more than one answer)	0. Uncertain income<br>1. Dislike to queue<br>2. Disappointed with the healthcare service<br>3. Disappointed with BPJS Kesehatan service<br>4. Difficult access to pay<br>5. Forget to pay<br>6. Others, mention	CODE: ☐☐☐
A10. If the reason is the income-uncertainty, what is your suggestion to solve the problem?		
A11. If the reason is the dislike to queue, what is your suggestion to solve the problem?		
A12. If the reason is the disappointment at the healthcare service, what is your suggestion to solve the problem?		
A13. If the reason is the disappointment at the BPJS Kesehatan service, what is your suggestion to solve the problem?		

A14. If the reason is the difficult access to pay, what is your suggestion to solve the problem?		
A15. If the reason is the forgetfulness, what is your suggestion to solve the problem?		
A16. Do you know if there are penalties for ignoring the payment of JKN/BPJS Kesehatan premium?<br><br>(What are the penalties ________________________)	0. No<br>1. Yes	CODE: ☐☐☐
A17. Do you have any Health Insurances other than JKN/BPJS Kesehatan?	0. No<br>1. Yes	CODE: ☐☐☐
A18. If yes, what health insurance do you have?	0. Jamkesmas/PBI<br>1. Jamkesda<br>2. Private	CODE: ☐☐☐
A19. Do you pay the insurance premium (A.18) regularly?	0. No<br>1. No premium<br>2. Yes	CODE: ☐☐☐

VI. ABILITY TO PAY

6.1. Average family expense for food

	TYPES OF EXPENSE	ONE PAST WEEK	ONE PAST MONTH
PM01	Vegetables and side dishes (IDR)	[ ][ ].[ ][ ][ ].[ ][ ][ ]	[ ][ ].[ ][ ][ ].[ ][ ][ ]
PM02	Fruits (IDR)	[ ][ ].[ ][ ][ ].[ ][ ][ ]	[ ][ ].[ ][ ][ ].[ ][ ][ ]
PM03	Water (IDR)	[ ][ ].[ ][ ][ ].[ ][ ][ ]	[ ][ ].[ ][ ][ ].[ ][ ][ ]
PM04	Staple food (IDR)	[ ][ ].[ ][ ][ ].[ ][ ][ ]	[ ][ ].[ ][ ][ ].[ ][ ][ ]
PM05	Cooking oil (IDR)	[ ][ ].[ ][ ][ ].[ ][ ][ ]	[ ][ ].[ ][ ][ ].[ ][ ][ ]
PM06	Spices (IDR)	[ ][ ].[ ][ ][ ].[ ][ ][ ]	[ ][ ].[ ][ ][ ].[ ][ ][ ]
PM07	Snack/softdrink (IDR)	[ ][ ].[ ][ ][ ].[ ][ ][ ]	[ ][ ].[ ][ ][ ].[ ][ ][ ]
PM08	Alcoholic drink (IDR)	[ ][ ].[ ][ ][ ].[ ][ ][ ]	[ ][ ].[ ][ ][ ].[ ][ ][ ]
	Total Expense for Food (IDR)	[ ][ ].[ ][ ][ ].[ ][ ][ ]	[ ][ ].[ ][ ][ ].[ ][ ][ ]

6.2. Average family expense for non-food

	TYPES OF EXPENSE	ONE PAST WEEK	ONE PAST MONTH
PN01	Rental House (if renting) (IDR)	[ ][ ].[ ][ ][ ].[ ][ ][ ]	[ ][ ].[ ][ ][ ].[ ][ ][ ]
PN02	Transport (IDR)	[ ][ ].[ ][ ][ ].[ ][ ][ ]	[ ][ ].[ ][ ][ ].[ ][ ][ ]
PN03	Cigarette/Tobacco (IDR)	[ ][ ].[ ][ ][ ].[ ][ ][ ]	[ ][ ].[ ][ ][ ].[ ][ ][ ]

	JENIS PENGELUARAN	SEBULAN TERAKHIR	SETAHUN TERAKHIR
PN04	Health (IDR)	[ ][ ].[ ][ ][ ].[ ][ ][ ]	[ ][ ].[ ][ ][ ].[ ][ ][ ]
PN05	Insurance Premium (IDR)	[ ][ ].[ ][ ][ ].[ ][ ][ ]	[ ][ ].[ ][ ][ ].[ ][ ][ ]
PN06	Education (IDR)	[ ][ ].[ ][ ][ ].[ ][ ][ ]	[ ][ ].[ ][ ][ ].[ ][ ][ ]
PN07	Entertainment (IDR)	[ ][ ].[ ][ ][ ].[ ][ ][ ]	[ ][ ].[ ][ ][ ].[ ][ ][ ]
PN08	Donation (IDR)	[ ][ ].[ ][ ][ ].[ ][ ][ ]	[ ][ ].[ ][ ][ ].[ ][ ][ ]
PN09	Electricity (IDR)	[ ][ ].[ ][ ][ ].[ ][ ][ ]	[ ][ ].[ ][ ][ ].[ ][ ][ ]
PN10	Water supply/PDAM (IDR)	[ ][ ].[ ][ ][ ].[ ][ ][ ]	[ ][ ].[ ][ ][ ].[ ][ ][ ]
PN11	Kerosene/firewood/LPG gas (IDR)	[ ][ ].[ ][ ][ ].[ ][ ][ ]	[ ][ ].[ ][ ][ ].[ ][ ][ ]
PN12	Telephone/credit/internet (IDR)	[ ][ ].[ ][ ][ ].[ ][ ][ ]	[ ][ ].[ ][ ][ ].[ ][ ][ ]
PN13	Household needs (bath soap, washing soap, toothbrush, shampoo, make up, perfume dsb)	[ ][ ].[ ][ ][ ].[ ][ ][ ]	[ ][ ].[ ][ ][ ].[ ][ ][ ]
PN14	Clothing (IDR)	[ ][ ].[ ][ ][ ].[ ][ ][ ]	[ ][ ].[ ][ ][ ].[ ][ ][ ]
PN15	Tax (IDR)	[ ][ ].[ ][ ][ ].[ ][ ][ ]	[ ][ ].[ ][ ][ ].[ ][ ][ ]
PN16	Housemaid/Driver (IDR)	[ ][ ].[ ][ ][ ].[ ][ ][ ]	[ ][ ].[ ][ ][ ].[ ][ ][ ]
PN17	Others (IDR), mention:	[ ][ ].[ ][ ][ ].[ ][ ][ ]	[ ][ ].[ ][ ][ ].[ ][ ][ ]
	Total Expense for Non-Food (IDR)	[ ][ ].[ ][ ][ ].[ ][ ][ ]	[ ][ ].[ ][ ][ ].[ ][ ][ ]

VII. WILLINGNESS TO PAY

KM.01	BPJS premium per person that is currently paid (IDR)	[ ][ ]. [ ][ ][ ]
KM.02	How do you think of the current BPJS premium?	0. Not affordable<br>1. Affordable<br>CODE: [ ][ ][ ]

7.1. IF AFFORDABLE

KM.03	If the premium is increased to IDR 28,000,-, are you still willing to pay JKN/BPJS Kesehatan regularly?	1. Yes<br>0. No<br>CODE: [ ][ ][ ]
KM.04	If the premium is increased to IDR 30,500,-, are you still willing to pay JKN/BPJS Kesehatan regularly?	1. Yes<br>0. No<br>CODE: [ ][ ][ ]
KM.05	If the premium is increased to IDR 33,000,-, are you still willing to pay JKN/BPJS Kesehatan regularly?	1. Yes<br>0. No<br>CODE: [ ][ ][ ]
KM.06	If the premium is increased to IDR 35,500,-, are you still willing to pay JKN/BPJS Kesehatan regularly?	1. Ya<br>0. Tidak<br>CODE: [ ][ ][ ]
KM.07	If the premium is increased to IDR 38,000,-, are you still willing to pay JKN/BPJS Kesehatan regularly?	1. Yes<br>0. No<br>CODE: [ ][ ][ ]
KM.08	If the premium is increased to IDR 40,500,-, are you still willing to pay JKN/BPJS Kesehatan regularly?	1. Yes<br>0. No<br>CODE: [ ][ ][ ]
KM.09	If the premium is increased to IDR 43,000,-, are you still willing to pay JKN/BPJS Kesehatan regularly?	1. Yes<br>0. No<br>CODE: [ ][ ][ ]

KM.10	If the premium is increased to IDR 45,500,-, are you still willing to pay JKN/BPJS Kesehatan regularly?	1. Yes<br>0. No	CODE: ☐☐☐
KM.11	If the premium is increased to IDR 48,000,-, are you still willing to pay JKN/BPJS Kesehatan regularly?	1. Yes<br>0. No	CODE: ☐☐☐
**7.2. IF NOT AFFORDABLE**			
KM.12	If the premium is reduced to IDR 23,000,-, are you willing to pay JKN/BPJS Kesehatan regularly?	1. Yes<br>0. No	CODE: ☐☐☐
KM.13	If the premium is reduced to IDR 20,500,-, are you willing to pay JKN/BPJS Kesehatan regularly?	1. Yes<br>0. No	CODE: ☐☐☐
KM.14	If the premium is reduced to IDR 18,000,-, are you willing to pay JKN/BPJS Kesehatan regularly?	1. Yes<br>0. No	CODE: ☐☐☐
KM.15	If the premium is reduced to IDR 15,500,-, are you willing to pay JKN/BPJS Kesehatan regularly?	1. Yes<br>0. No	CODE: ☐☐☐
KM.16	If the premium is reduced to IDR 13,000,-, are you willing to pay JKN/BPJS Kesehatan regularly?	1. Yes<br>0. No	CODE: ☐☐☐
KM.17	If the premium is reduced to IDR 10,500,-, are you willing to pay JKN/BPJS Kesehatan regularly?	1. Yes<br>0. No	CODE: ☐☐☐
KM.18	If the premium is reduced to IDR 8,000,-, are you willing to pay JKN/BPJS Kesehatan regularly?	1. Yes	CODE: ☐☐☐

		0. No	
KM.19	If the premium is reduced to IDR 5,500,-, are you willing to pay JKN/BPJS Kesehatan regularly?	1. Yes<br>1. No	CODE: ☐☐☐
KM.20	If the premium is reduced to IDR 3,000,-, are you willing to pay JKN/BPJS Kesehatan regularly?	2. Yes<br>1. No	CODE: ☐☐☐
KM.21	If the premium is eliminated (IDR 0), are you willing to apply as a BPJS participant?	2. Yes<br>0. No	CODE: ☐☐☐

VIII. PARTICIPANT SATISFACTION

Have you ever used the following healthcare facilities by employing BPJS/JKN KIS card?

And how do you rate the service provided by the healthcare facilities?

		Very Dissatisfied	Dissatisfied	Neutral	Satisfied	Very Satisfied
K.01	Puskesmas (Community Health Center)	0	1	2	3	4
K.02	Family doctor	0	1	2	3	4
K.03	General doctor clinic	0	1	2	3	4
K.04	Hospital	0	1	2	3	4
K.05	Others, mention	0	1	2	3	4

Code K01-K05

0. Very dissatisfied
1. Dissatisfied
2. Neutral
3. Satisfied
4. Very satisfied

CORRECTION SHEET

No.	Variable Number	Problem	Note
