# Supplementary material for: Why did informal sector workers stop paying for health insurance in Indonesia? Exploring enrollees’ ability and willingness to pay
Source: PLoS One. 2021 Jun 4;16(6):e0252708. doi: 10.1371/journal.pone.0252708 (PMC8177660; doi:10.1371/journal.pone.0252708)
Supplement: S2 File — (PDF) [file pone.0252708.s003.pdf]

Pusat KP-MAK

PUSAT KEBIJAKAN PEMBIAYAAN DAN MANAJEMEN ASURANSI KESEHATAN
FAKULTAS KEDOKTERAN UNIVERSITAS GADJAH MADA

INSTRUMEN KUANTITATIF

Survey Kesehatan Rumah Tangga
Pusat KPMAK FK UGM

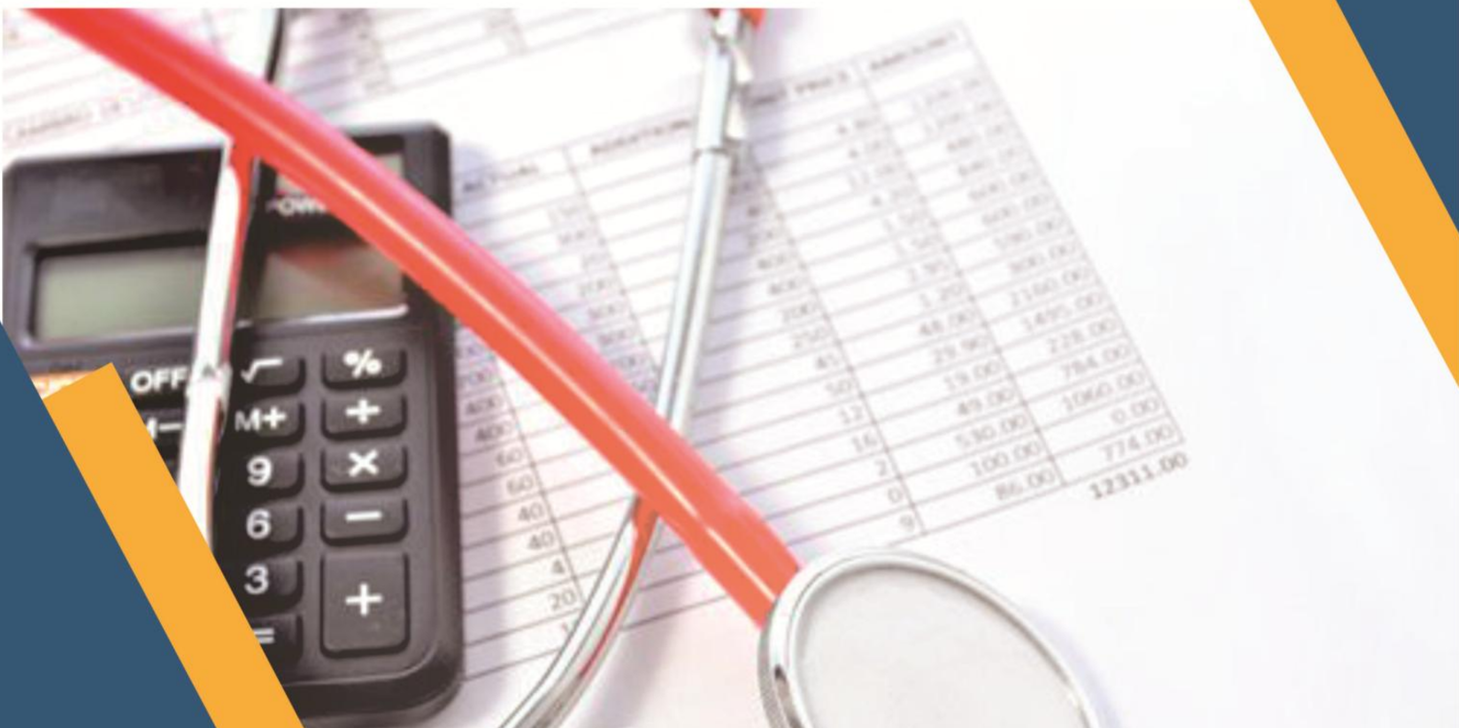

0274 - 631022 pusatkpmak@ugm.ac.id

Fakultas Kedokteran Universitas Gadjah Mada
Gedung Radioputro lt. 2, Sayap Barat
Jl. Farmako, Sekip Utara, D. I. Yogyakarta, Indonesia, 55281

Survey Kesehatan Rumah Tangga
Pusat KP-MAK FK UGM

Informed Consent

Kami Pusat KP-MAK FK UGM, saat ini sedang melakukan sebuah survey kesehatan rumah tangga. Untuk ini kami mohon kerjasama Bapak/Ibu untuk menjadi informan/responden pada penelitian ini dan dapat memberikan jawaban yang sesungguhnya dengan kondisi saat ini.

Keikutsertaan dalam wawancara ini bersifat sukarela, Bapak/Ibu dapat memilih untuk ikut serta atau tidak ikut serta dan tidak menjawab pertanyaan. Apabila Bapak/Ibu memutuskan untuk tidak turut serta, keputusan tersebut tidak akan berpengaruh apapun terhadap pelayanan/perlakuan yang seharusnya Bapak/Ibu terima. Kami meyakinkan pendapat dan keterangan dari Bapak/Ibu sangat berarti dan akan berguna bagi pembuatan kebijakan di Indonesia dan identitas serta informasi dari Bapak/Ibu akan kami rahasiakan, semua jenis keterangan yang Bapak/Ibu berikan tidak akan dibagi dengan pihak lain. Bapak/Ibu akan dicirikan dengan sebuah nomor Identitas responden yang merahasiakan data pribadi Bapak/Ibu.

Dengan menandatangani formulir ini, berarti Bapak/Ibu telah membaca keterangan di atas dan memahaminya, dan selanjutnya memutuskan untuk ikut serta tanpa paksaan dalam survey penelitian ini. Setelah menandatangani berkas ini, Bapak/Ibu dapat membatalkan keikutsertaan Anda dalam penelitian ini kapanpun tanpa akibat apapun.

NAMA Responden ____________________

Tanda tangan Responden ____________________

Tanggal ____________________

Atas kerja sama Bapak/Ibu, saya ucapkan banyak terima kasih.

Ttd. Pewawancara

Nama_________________

SURVEY KESEHATAN RUMAH TANGGA<br>PUSAT KP-MAK FK UGM		
KUESIONER		
NO RESPONDEN ☐☐☐	TANGGAL:	NAMA SURVEYOR:

I. KETERANGAN TEMPAT

KT01 Propinsi		KODE: ☐☐☐
KT02 Kabupaten/Kota		KODE: ☐☐☐
KT03 Kecamatan		
KT04 Desa/kelurahan		
KT05 RW		
KT06 Nomor Telepon/HP		

**Kode KT01**	**Kode KT02**		
12. Sumatera Utara	1212. Deli Serdang	5103. Badung	7102. Minahasa
14. Riau	1213. Langkat	5104. Gianyar	7106. Minahasa Utara
35. Jawa Timur	1216. Pakpak Bharat	5108. Buleleng	7171. Kota Manado
36. Banten	1401. Kuantan Singingi	5312. Ngada	7205. Donggala
51. Bali	1405. Siak	5314. Rote Ndao	7208. Tojo Una Una
53. Nusa Tenggara Timur	1473. Kota Dumai	5371. Kota Kupang	7271. Kota Palu
61. Kalimantan Barat	3529. Sumenep	6101. Sambas	9419. Sarmi
64. Kalimantan Timur	3576. Kota Mojokerto	6112. Kubu Raya	9420. Kerom
71. Sulawesi Utara	3578. Kota Surabaya	6172. Kota Singkawang	9471. Kota Jayapura
72. Sulawesi Tengah	3601. Pandeglang	6405. Berau	9105. Kota Manokwari
91. Papua Barat	3604. Serang	6409. Penajam Paser Utara	9108. Raja Ampat
94. Papua	3674. Kota Tangerang Selatan	6472. Kota Samarinda	9171. Kota Sorong

II. IDENTITAS RESPONDEN

IR01 Usia responden		
IR02 Jenis kelamin		KODE: ☐☐☐
IR03 Pendidikan terakhir		KODE: ☐☐☐
IR04 Pekerjaan utama kepala rumah tangga		KODE: ☐☐☐
IR05 Pekerjaan istri		KODE: ☐☐☐
IR06 Rerata pendapatan keluarga per bulan<br>a. Suami (Rp)	[ ][ ].[ ][ ][ ].[ ][ ][ ]	

b. Istri (Rp)<br><br>c. Anggota keluarga lainnya (Rp)	[ ][ ].[ ][ ][ ].[ ][ ][ ]<br><br>[ ][ ].[ ][ ][ ].[ ][ ][ ]	
IR07 Jumlah anggota keluarga		
IR08 Keberadaan anak sekolah<br><br>a. Anak ke 1<br><br>b. Anak ke 2<br><br>c. Anak ke 3<br><br>d. Anak ke 4	0. Tidak ada 1. Ada<br><br>Tingkat:<br>Kelas:<br><br>Tingkat:<br>Kelas:<br><br>Tingkat:<br>Kelas:<br><br>Tingkat:<br>Kelas:	KODE: ☐☐☐
IR09 Status kepemilikan rumah	0. Kontrak/Sewa<br>1. Milik sendiri<br>2. Rumah dinas<br>3. Milik pemerintah<br>4. Lainnya, sebutkan	KODE: ☐☐☐
IR10 Dalam keluarga Anda, berapa orang yang telah memiliki kartu JKN/BPJS Kesehatan	0. Seorang<br>1. Dua orang<br>2. Tiga orang<br>3. Empat orang<br>4. Lima orang<br>5. Enam orang<br>6. Lebih dari enam orang	KODE: ☐☐☐

**Kode IR02**<br>0. Laki-laki<br>1. Perempuan<br><br>**Kode IR03**<br>0. Tidak tamat SD<br>1. Tamat SD<br>2. Tamat SMP<br>3. Tamat SMA<br>4. D1/D2/D3<br>5. S1	6. S2<br>7. S3<br><br>**Kode IR04**<br>0. Kaki lima<br>1. Buruh<br>2. Pensiunan<br>3. Tani<br>4. Nelayan<br>5. Tukang<br>6. Honorer	7. Pegawai swasta<br>8. Wiraswasta/Pedagang<br>9. Ibu rumah tangga<br>10. Pelajar/Mahasiswa<br>11. Rohaniwan/Rohaniwati<br>12. Tidak bekerja<br>13. Lain-lain<br><br>**Kode IR05**<br>0. Kaki lima<br>1. Buruh	2. Pensiunan<br>3. Tani<br>4. Nelayan<br>5. Tukang<br>6. Honorer<br>7. Pegawai swasta<br>8. Wiraswasta/Pedagang<br>9. Ibu rumah tangga<br>10. Pelajar/Mahasiswa<br>11. Rohaniwan/Rohaniwati<br>12. Tidak bekerja<br>13. Lain-lain

III. PERKIRAAN BEBAN TANGGUNGAN KESEHATAN

Dalam satu bulan, berapa kali rata-rata anggota keluarga pergi rawat jalan di:		
TK01 Puskesmas	0. Tidak pernah<br>1. Satu kali<br>2. Dua kali<br>3. Tiga kali<br>4. Lebih dari tiga kali	KODE: ☐☐☐
TK02 Klinik	0. Tidak pernah<br>1. Satu kali	KODE: ☐☐☐

	2. Dua kali<br>3. Tiga kali<br>4. Lebih dari tiga kali	
TK03 Rumah sakit	5. Tidak pernah<br>6. Satu kali<br>7. Dua kali<br>8. Tiga kali<br>9. Lebih dari tiga kali	KODE: ☐☐☐
Dalam satu tahun, berapa kali rata-rata anggota keluarga rawat inap di:		
TK04 Puskesmas	0. Tidak pernah<br>1. Satu kali<br>2. Dua kali<br>3. Tiga kali<br>4. Lebih dari tiga kali	KODE: ☐☐☐
TK05 Klinik	0. Tidak pernah<br>1. Satu kali<br>2. Dua kali<br>3. Tiga kali<br>4. Lebih dari tiga kali	KODE: ☐☐☐
TK06 Rumah sakit	0. Tidak pernah<br>1. Satu kali<br>2. Dua kali<br>3. Tiga kali<br>4. Lebih dari tiga kali	KODE: ☐☐☐
Dalam satu bulan terakhir, berapa kali rata-rata anggota keluarga melakukan pengobatan di luar fasilitas kesehatan:		
TK07 Membeli obat di warung/toko obat	0. Tidak pernah<br>1. Satu kali<br>2. Dua kali<br>3. Tiga kali<br>4. Lebih dari tiga kali	KODE: ☐☐☐
TK08 Pengobatan alternatif	0. Tidak pernah<br>1. Satu kali<br>2. Dua kali<br>3. Tiga kali<br>4. Lebih dari tiga kali	KODE: ☐☐☐

IV. PENANGGULANGAN RISIKO

PR01 Berdasarkan pengalaman, ketika ada anggota keluarga yang sakit, bagaimana cara Anda menanggung biayanya?	0. biaya sendiri<br>1. dibantu saudara-saudara<br>2. dibantu tetangga<br>3. dibantu kantor/organisasi<br>4. dibantu pemerintah<br>5. ikut menjadi peserta jaminan/asuransi kesehatan<br>6. Lain-lain, sebutkan	KODE: ☐☐☐
PR02 Terkait dengan biaya, bagaimana perasaan Anda jika sewaktu-waktu ada anggota keluarga yang sakit dan memerlukan biaya perawatan di rumah sakit?	0. saya sangat tidak khawatir<br>1. saya tidak khawatir<br>2. saya agak khawatir<br>3. saya sangat khawatir	KODE: ☐☐☐
PR03 Untuk mengantisipasi jika sewaktu-waktu ada anggota keluarga yang sakit, apa yang akan Anda lakukan?	0. menabung untuk biaya kesehatan<br>1. menjual barang<br>2. meminjam uang<br>3. mengharapkan bantuan<br>4. ikut asuransi kesehatan<br>5. belum terpikirkan sama sekali<br>6. lain-lain, sebutkan	KODE: ☐☐☐

V. ASURANSI

A01. Apakah Anda memiliki kartu BPJS Kesehatan?	0. Tidak<br>1. Ya	KODE: ☐☐☐
A02. Jika ya, untuk kelas apa?<br>(Jika tidak, langsung ke pertanyaan A17.)	0. Kelas I<br>1. Kelas II<br>2. Kelas III	KODE: ☐☐☐
A03. Jika Anda memilih kelas III, apa alasannya?<br>(Hanya untuk yang menjawab pertanyaan A02: kelas III)	0. Preminya paling murah/yang paling terjangkau<br>1. Waktu tunggunya lebih pendek<br>2. Ikut-ikutan tetangga/teman<br>3. Lainnya, sebutkan	KODE: ☐☐☐
A04. Apa alasan Anda mendaftar menjadi peserta JKN/BPJS?	0. Program pemerintah/ diwajibkan oleh pemerintah<br>1. Ada anggota keluarga yang sakit<br>2. Mengantisipasi jika ada anggota keluarga yang sakit<br>3. Ikut-ikutan tetangga/teman<br>4. Lainnya, sebutkan	KODE: ☐☐☐
A05. Dari mana Anda mendapatkan info tentang JKN/BPJS?	0. Penjelasan BPJS<br>1. Perangkat pemerintah<br>2. Media massa<br>3. Teman/tetangga<br>4. Lainnya, sebutkan	KODE: ☐☐☐
A06. Pada tahun berapa Anda mulai terdaftar sebagai peserta JKN/BPJS Kesehatan?	0. 2014<br>1. 2015<br>2. 2016	KODE: ☐☐☐
A07. Apakah Anda rutin membayar iuran JKN/BPJS Kesehatan?	0. Tidak<br>1. Ya	KODE: ☐☐☐
A08. Jika ya, apa alasan Anda?<br>(Lansung ke pertanyaan A16.)	0. Sudah kewajiban<br>1. Supaya tidak terkena sanksi<br>2. Supaya bisa mengakses pelayanan kesehatan/ kebaikan responden<br>3. Lainnya, sebutkan	KODE: ☐☐☐
A09. Jika tidak, apa alasan Anda?<br>(Jawaban bisa lebih dari satu)	0. Pendapatan tidak menentu<br>1. Malas mengantri<br>2. Kecewa terhadap pelayanan fasilitas kesehatan<br>3. Kecewa terhadap pelayanan BPJS Kesehatan<br>4. Kesulitan akses pembayaran<br>5. Lupa membayar<br>6. Lainnya, sebutkan	KODE: ☐☐☐
A10. Jika alasannya pendapatan yang tidak menentu, apa usulan Anda untuk mengatasi masalah tersebut?		
A11. Jika alasannya malas mengantri, apa usulan Anda untuk mengatasi masalah tersebut?		

A12. Jika alasannya kecewa terhadap pelayanan fasilitas kesehatan, apa usulan Anda untuk mengatasi masalah tersebut?		
A13. Jika alasannya kecewa terhadap pelayanan BPJS Kesehatan, apa usulan Anda untuk mengatasi masalah tersebut?		
A14. Jika alasannya kesulitan akses pembayaran, apa usulan Anda untuk mengatasi masalah tersebut?		
A15. Jika alasannya lupa membayar, apa usulan Anda untuk mengatasi masalah tersebut?		
A16. Apakah Anda tahu jika ada sanksi bagi siapa saja yang menunggak pembayaran iuran JKN/BPJS Kesehatan<br><br>(Seperti apa contoh sanksinya ________________________)	0. Tidak<br>1. Ya	KODE: ☐☐☐
A17. Apakah Anda memiliki asuransi/jaminan kesehatan selain JKN/BPJS Kesehatan?	0. Tidak<br>1. Ya	KODE: ☐☐☐
A18. Jika ya, apa asuransi yang Anda miliki/gunakan?	0. Jamkesmas/PBI<br>1. Jamkesda<br>2. Swasta	KODE: ☐☐☐
A19. Apakah Anda membayar iuran asuransi tersebut (A.18) secara rutin?	0. Tidak<br>1. Tidak ada iuran<br>2. Ya	KODE: ☐☐☐

VI. KEMAMPUAN MEMBAYAR

6.1.Rata-rata pengeluaran keluarga untuk makanan

	JENIS PENGELUARAN	SEMINGGU TERAKHIR	SEBULAN TERAKHIR
PM01	Sayur dan lauk pauk (Rp)	[ ][ ].[ ][ ][ ].[ ][ ][ ]	[ ][ ].[ ][ ][ ].[ ][ ][ ]
PM02	Buah-buahan (Rp)	[ ][ ].[ ][ ][ ].[ ][ ][ ]	[ ][ ].[ ][ ][ ].[ ][ ][ ]
PM03	Air minum (Rp)	[ ][ ].[ ][ ][ ].[ ][ ][ ]	[ ][ ].[ ][ ][ ].[ ][ ][ ]
PM04	Makanan pokok (Rp)	[ ][ ].[ ][ ][ ].[ ][ ][ ]	[ ][ ].[ ][ ][ ].[ ][ ][ ]
PM05	Minyak goreng (Rp)	[ ][ ].[ ][ ][ ].[ ][ ][ ]	[ ][ ].[ ][ ][ ].[ ][ ][ ]
PM06	Bumbu dapur (Rp)	[ ][ ].[ ][ ][ ].[ ][ ][ ]	[ ][ ].[ ][ ][ ].[ ][ ][ ]
PM07	Makanan kecil/Jajanan/Minuman ringan (Rp)	[ ][ ].[ ][ ][ ].[ ][ ][ ]	[ ][ ].[ ][ ][ ].[ ][ ][ ]
PM08	Minuman beralkohol (Rp)	[ ][ ].[ ][ ][ ].[ ][ ][ ]	[ ][ ].[ ][ ][ ].[ ][ ][ ]
	Total Pengeluaran Makanan (Rp)	[ ][ ].[ ][ ][ ].[ ][ ][ ]	[ ][ ].[ ][ ][ ].[ ][ ][ ]

6.2.Rata-rata pengeluaran keluarga untuk non-makanan

	JENIS PENGELUARAN	SEBULAN TERAKHIR	SETAHUN TERAKHIR
PN01	Sewa rumah (bila masih sewa) (Rp)	[ ][ ].[ ][ ][ ].[ ][ ][ ]	[ ][ ].[ ][ ][ ].[ ][ ][ ]
PN02	Transportasi (Rp)	[ ][ ].[ ][ ][ ].[ ][ ][ ]	[ ][ ].[ ][ ][ ].[ ][ ][ ]
PN03	Rokok/Tembakau (Rp)	[ ][ ].[ ][ ][ ].[ ][ ][ ]	[ ][ ].[ ][ ][ ].[ ][ ][ ]

	JENIS PENGELUARAN	SEBULAN TERAKHIR	SETAHUN TERAKHIR
PN04	Kesehatan (Rp)	[ ][ ] . [ ][ ][ ]. [ ][ ][ ]	[ ][ ] . [ ][ ][ ]. [ ][ ][ ]
PN05	Premi asuransi (Rp)	[ ][ ] . [ ][ ][ ]. [ ][ ][ ]	[ ][ ] . [ ][ ][ ]. [ ][ ][ ]
PN06	Pendidikan (Rp)	[ ][ ] . [ ][ ][ ]. [ ][ ][ ]	[ ][ ] . [ ][ ][ ]. [ ][ ][ ]
PN07	Rekreasi/Hiburan (Rp)	[ ][ ] . [ ][ ][ ]. [ ][ ][ ]	[ ][ ] . [ ][ ][ ]. [ ][ ][ ]
PN08	Sumbangan/Iuran (Rp)	[ ][ ] . [ ][ ][ ]. [ ][ ][ ]	[ ][ ] . [ ][ ][ ]. [ ][ ][ ]
PN09	Listrik (Rp)	[ ][ ] . [ ][ ][ ]. [ ][ ][ ]	[ ][ ] . [ ][ ][ ]. [ ][ ][ ]
PN10	Air/PDAM (Rp)	[ ][ ] . [ ][ ][ ]. [ ][ ][ ]	[ ][ ] . [ ][ ][ ]. [ ][ ][ ]
PN11	Minyak tanah/kayu bakar/gas LPG (Rp)	[ ][ ] . [ ][ ][ ]. [ ][ ][ ]	[ ][ ] . [ ][ ][ ]. [ ][ ][ ]
PN12	Telepon/pulsa/internet (Rp)	[ ][ ] . [ ][ ][ ]. [ ][ ][ ]	[ ][ ] . [ ][ ][ ]. [ ][ ][ ]
PN13	Kebutuhan rumah tangga (Sabun mandi, sabun cuci, sikat gigi, sampo, make up, parfum dsb)	[ ][ ] . [ ][ ][ ]. [ ][ ][ ]	[ ][ ] . [ ][ ][ ]. [ ][ ][ ]
PN14	Pakaian (Rp)	[ ][ ] . [ ][ ][ ]. [ ][ ][ ]	[ ][ ] . [ ][ ][ ]. [ ][ ][ ]
PN15	Pajak (Rp)	[ ][ ] . [ ][ ][ ]. [ ][ ][ ]	[ ][ ] . [ ][ ][ ]. [ ][ ][ ]
PN16	Pembantu/Supir (Rp)	[ ][ ] . [ ][ ][ ]. [ ][ ][ ]	[ ][ ] . [ ][ ][ ]. [ ][ ][ ]
PN17	Lain-lain (Rp), sebutkan:	[ ][ ] . [ ][ ][ ]. [ ][ ][ ]	[ ][ ] . [ ][ ][ ]. [ ][ ][ ]
	Total Pengeluaran Non-Makanan (Rp)	[ ][ ] . [ ][ ][ ]. [ ][ ][ ]	[ ][ ] . [ ][ ][ ]. [ ][ ][ ]

VII. KEMAUAN MEMBAYAR

KM.01	Biaya premi BPJS per orang yang dibayarkan saat ini (Rp)	[ ] [ ]. [ ] [ ] [ ]
KM.02	Bagaimana pendapat Bapak/Ibu terhadap besaran premi BPJS saat ini	0. Tidak terjangkau<br>1. Terjangkau<br>KODE: ☐☐☐

7.1. JIKA TERJANGKAU

KM.03	Jika biaya dinaikkan menjadi Rp 28.000,-, apakah Anda masih bersedia membayar iuran secara rutin?	1. Ya<br>0. Tidak<br>KODE: ☐☐☐
KM.04	Jika biaya dinaikkan menjadi Rp 30.500,- , apakah Anda masih bersedia membayar iuran secara rutin?	1. Ya<br>0. Tidak<br>KODE: ☐☐☐
KM.05	Jika biaya dinaikkan menjadi Rp 33.000,-, apakah Anda masih bersedia membayar iuran secara rutin?	1. Ya<br>0. Tidak<br>KODE: ☐☐☐
KM.06	Jika biaya dinaikkan menjadi Rp 35.500,-, apakah Anda masih bersedia membayar iuran secara rutin?	1. Ya<br>0. Tidak<br>KODE: ☐☐☐
KM.07	Jika biaya dinaikkan menjadi Rp 38.000,-, apakah Anda masih bersedia membayar iuran secara rutin?	1. Ya<br>0. Tidak<br>KODE: ☐☐☐
KM.08	Jika biaya dinaikkan menjadi Rp 40.500,-, apakah Anda masih bersedia membayar iuran secara rutin?	1. Ya<br>0. Tidak<br>KODE: ☐☐☐
KM.09	Jika biaya dinaikkan menjadi Rp 43.000,-, apakah Anda masih bersedia membayar iuran secara rutin?	1. Ya<br>0. Tidak<br>KODE: ☐☐☐

KM.10	Jika biaya dinaikkan menjadi Rp 45.500,-, apakah Anda masih bersedia membayar iuran secara rutin?	1. Ya<br>0. Tidak	KODE: ☐☐☐
KM.11	Jika biaya dinaikkan menjadi Rp 48.000,-, apakah Anda masih bersedia membayar iuran secara rutin?	1. Ya<br>0. Tidak	KODE: ☐☐☐
**7.2. JIKA TIDAK TERJANGKAU**			
KM.12	Jika biaya diturunkan menjadi Rp 23.000,-, apakah Anda akan membayar iuran JKN/BPJS Kesehatan secara rutin?	1. Ya<br>0. Tidak	KODE: ☐☐☐
KM.13	Jika biaya diturunkan menjadi Rp 20.500,-, apakah Anda akan membayar iuran JKN/BPJS Kesehatan secara rutin?	1. Ya<br>0. Tidak	KODE: ☐☐☐
KM.14	Jika biaya diturunkan menjadi Rp 18.000,-, apakah Anda akan membayar iuran JKN/BPJS secara rutin?	1. Ya<br>0. Tidak	KODE: ☐☐☐
KM.15	Jika biaya diturunkan menjadi Rp 15.500,-, apakah Anda akan membayar iuran JKN/BPJS secara rutin?	1. Ya<br>0. Tidak	KODE: ☐☐☐
KM.16	Jika biaya diturunkan menjadi Rp 13.000,-, apakah Anda akan membayar iuran JKN/BPJS secara rutin?	1. Ya<br>0. Tidak	KODE: ☐☐☐
KM.17	Jika biaya diturunkan menjadi Rp 10.500,-, apakah Anda akan membayar iuran JKN/BPJS secara rutin?	1. Ya<br>0. Tidak	KODE: ☐☐☐
KM.18	Jika biaya diturunkan menjadi Rp 8.000,-, apakah Anda akan membayar iuran JKN/BPJS secara rutin?	1. Ya	KODE: ☐☐☐

		0. Tidak
KM.19	Jika biaya diturunkan menjadi Rp 5.500,-, apakah Anda akan membayar iuran JKN/BPJS secara rutin?	1. Ya<br>1. Tidak<br>KODE: ☐☐☐
KM.20	Jika biaya diturunkan menjadi Rp 3.000,-, apakah Anda akan membayar iuran JKN/BPJS secara rutin?	2. Ya<br>1. Tidak<br>KODE: ☐☐☐
KM.21	Jika biaya tersebut ditiadakan (Rp 0) apakah Anda bersedia menjadi anggota BPJS?	2. Ya<br>0. Tidak<br>KODE: ☐☐☐

VIII. KEPUASAN PESERTA

Apakah Anda pernah memanfaatkan fasilitas kesehatan di bawah ini dengan menggunakan kartu BPJS/JKN KIS?

Dan bagaimana penilaian anda terhadap pelayanan yang diberikan di fasilitas kesehatan tersebut?

		Sangat Tidak Puas	Tidak Puas	Biasa Saja	Puas	Sangat Puas
K.01	Puskesmas	0	1	2	3	4
K.02	Dokter keluarga	0	1	2	3	4
K.03	Klinik dokter umum	0	1	2	3	4
K.04	Rumah sakit	0	1	2	3	4
K.05	Lainnya, sebutkan	0	1	2	3	4

Kode K01-K05
0. Sangat tidak puas
1. Tidak puas
2. Biasa saja
3. Puas
4. Sangat puas

LEMBAR KOREKSI

No.	Nomor Variabel	Masalah	Catatan
