# Supplementary material for: Why did informal sector workers stop paying for health insurance in Indonesia? Exploring enrollees’ ability and willingness to pay
Source: PLoS One. 2021 Jun 4;16(6):e0252708. doi: 10.1371/journal.pone.0252708 (PMC8177660; doi:10.1371/journal.pone.0252708)
Supplement: S3 File — (DOCX) [file pone.0252708.s004.docx]

# Qualitative questions ATP/WTP Indonesian

# PEDOMAN WAWANCARA PADA PESERTA

Kami Pusat KP-MAK FK UGM, saat ini sedang melakukan kajian terkait bagimana menyediakan Asuransi Sosial kepada masyarakat . Untuk ini kami mohon kerjasama Ibu/Bapak/Saudara untuk menjadi informan/responden pada penelitian ini dan dapat memberikan jawaban yang sesungguhnya dengan kondisi saat ini.

Keikutsertaan dalam wawancara ini bersifat sukarela, Ibu/Bapak/Saudara dapat memilih untuk ikut serta atau tidak ikut serta dan tidak menjawab pertanyaan. Apabila Ibu/Bapak/Saudara memutuskan untuk tidak turut serta,

Kami meyakinkan pendapat dan keterangan dari Ibu/Bapak/Saudara sangat berarti dan akan berguna bagi pembuatan kebijakan di Indonesia dan identitas serta informasi dari Ibu/Bapak/Saudara akan kami rahasiakan, semua jenis keterangan yang Ibu/Bapak/Saudara berikan tidak akan diberikan pada pihak lain.

Atas kerja sama Ibu/Bapak/Saudara, saya ucapkan banyak terima kasih.

# PROFILE RESPONDEN

1. Nama pewawancara:
2. Nama responden :
3. Jenis kelamin :
4. Usia :
5. Pekerjaan :
6. Tanggal wawancara :

# PERTANYAAN WAWANCARA

1. Apakah sebelumnya Anda atau keluarga Anda pernah membeli asuransi jenis apapun, misalnya untuk rumah, sepeda motor, atau asuransi jiwa?
2. Mengapa Anda tertarik untuk mendaftar BPJS Kesehatan?

Jika tanggapannya sangat umum, misalnya, "ikut-ikutan", telusuri untuk memahami mendapat pengaruh dari siapa.

1. Sudah berapa bulan Anda membayar premi? Dan sistem apa yang Anda gunakan untuk membayar premi ? Apakah Anda merasa mudah dan nyaman untuk membayar setiap bulannya ?

Jika tidak: Apakah ada metode pembayaran lain dan / atau jadwal pembayaran yang lebih berpengaruh untuk Anda? Apa yang Anda sarankan?

Jika sistem selain transfer bank / ATM: Apakah Anda memiliki rekening bank?

1. Apakah Anda pernah memanfaatkan layanan Program JKN-KIS?

Jika ya :

- Layanan apa yang Anda gunakan?

(rawat jalan / rawat inap, FKTP atau FKRTL)

- Apakah Anda mendapatkan obat secara gratis?
- Ketika Anda telah memiliki JKN, apakah Anda pernah membayar layanan kesehatan menggunakan uang pribadi Anda?

Jika ya, mengapa pada saat itu Anda tidak menggunakan JKN ?

- Bagaimana pendapat Anda tentang layanan program JKN-KIS? Menurut Anda apa yang harus diubah atau ditingkatkan?

1. Menurut Anda, apa keuntungan dan kerugian dari asuransi? Dan siapa yang seharusnya mendaftar di JKN ?
2. Mengapa Anda berhenti membayar iuran/premi JKN?
3. Jika alasannya tidak mampu, apa yang berubah dalam kondisi Anda, sehingga Anda berubah dari mampu membayar menjadi tidak mampu membayar?
4. Jika alasannya tidak mampu, mengapa Anda tidak ditanggung oleh PBI yang seharusnya memberikan JKN bagi orang yang tidak mampu?
5. Jika ada yang menawarkan asuransi selain BPJS seperti asuransi swasta, apakah Anda berminat ?
6. Apakah Anda pernah sakit sejak Anda berhenti membayar iuran JKN? Apa yang Anda lakukan saat itu? Berapa biayanya yang Anda keluarkan?
7. Apa alasan Anda membayar kembali iuran BPJS Kesehatan ?
