# Supplementary material for: Why did informal sector workers stop paying for health insurance in Indonesia? Exploring enrollees’ ability and willingness to pay
Source: PLoS One. 2021 Jun 4;16(6):e0252708. doi: 10.1371/journal.pone.0252708 (PMC8177660; doi:10.1371/journal.pone.0252708)
Supplement: S4 File — (DOCX) [file pone.0252708.s005.docx]

Qualitative follow up questions

1. Have you or any of your family ever bought any kind of insurance before, for example for your house, or motorbike, or life insurance?

2. Why were you interested to register BPJS Kesehatan ?

If the response is very general, e.g. "ikut-ikutan", follow up to understand who the

influencers were.

3. How many months did you pay premiums for? And what system did you use to pay the premium? Did you find it easy and convenient to pay every month?

If no: Is there some other payment method and/or payent schedule that would work

better for you? What would you suggest?

If system other than bank transfer/ATM): do you have a bank account?

4. Have you ever utilized the service of JKN-KIS Program?

If yes, what did you use it for? (outpatient/inpatient, FKTP or FKRTL) Did you get your medicines for free?

When you had JKN, did you ever pay for any health services out of your own pocket?

Which ones, and why did you not use JKN that time?

How do you think about the service of JKN-KIS program? What do you think should be changed or improved?

5. What do you think are the advantages and disadvantages of insurance? Who do you think

should sign up for JKN?

6. Why did you stop paying the JKN premium?

1. If the reason is not able, what changed in your circumstances, so that you went from being able to pay to being not able to pay?
2. If the reason is not able, why are you not covered by PBI, which is supposed to provide JKN for people who cannot afford to pay?
3. If I were to offer to sign you up right now for a regular payment of xxx. would you sign up?
4. Have you been sick at all since you stopped paying your JKN premiums? What did you do on that occasion? How much did it cost you?

7. What reasons that will make you pay back premium of BPJS Kesehatan ?
