# Supplementary material for: Why did informal sector workers stop paying for health insurance in Indonesia? Exploring enrollees’ ability and willingness to pay
Source: PLoS One. 2021 Jun 4;16(6):e0252708. doi: 10.1371/journal.pone.0252708 (PMC8177660; doi:10.1371/journal.pone.0252708)
Supplement: Attachment — Submitted filename: Response to Reviewers.docx [file pone.0252708.s006.docx]

**The authors would like to thank the reviewers for their thorough reading of the paper, useful comments and constructive suggestions, which have certainly helped in improving the paper.**

We checked the PLOS style requirements, and have included a copy of our questionnaires and interview guide in both Indonesian and English as supporting information. Inspired by the COREQ, we have added some more information about those who conducted the qualitative interviews.

Reviewer #1: This paper addressed one of very critical issues of the health insurance coverage among informal sector from a unique angle. The topic and results of this study could be very valuable for JKN development as well as the development of health insurance schemes in other LMICs.

Here are a few recommendations for the potential improvement.

1. Can author add the sections regarding the literature review and theories related to this study? ATP and WTP have been studied intensively. It would be great that authors can show how these existing knowledges guide the design and analysis of this study. For example, we understand that people’s health state will be an important factor affecting their WTP and actual enrollment of insurance significantly.
**Thank you for this comment. We agree with this suggestion and have added several recent articles to the Introduction. While this is not an extensive literature review, the research design has been informed by our understanding of the relevant literature, and the aims of the implementation of JKN in 2014.**

2. At the end of the paper, authors made some very useful policy recommendations. It would be great if authors can make better connections between the results of study and these recommendations, with the considerations of feasibilities of their implementation in JKN.
**Thank you for this suggestion. In the Discussion, we have explicitly tried to connect each of the recommendations to the results that we present. To keep the formulation of the recommendations clear and concise, we prefer not to further elaborate on these given the space already allocated to recommendations in the Discussion.**

3. In line 124, It is stated that “ATP was calculated as five percent of a household’s non- subsistence expenditure, i.e., less the amount spent on food”; while in line 127, it is stated that “it is still half of the most stringent measure of catastrophic health expenditure”. To my knowledge, these two are two different measurements, and please make sure their compatibilities.

**Many thanks for pointing this out. We have tried to clarify the text. The reference to catastrophic health expenditure is related to the five percent cut off. The line in 127 is justifying the selection of a five percent cut off: as well as being a ‘typical’ percentage of household income spent on healthcare in LMICs, it is also less than half the most stringent estimation of catastrophic health expenditure (i.e., 10% of income used on healthcare). This section has been edited to clarify this meaning.**

Reviewer #2: Referee Report PONE-D-30634
Ability and willingness to pay for National Health Insurance in Indonesia: a study of the informal sector.

The iterative bidding approach (Randall et al., 1974) starts by querying individuals at some initial dollar value and keeps raising (or lowering) the value until the respondent declines (accepts) to pay. This final dollar amount is interpreted as the respondent's WTP. However, this approach has been virtually abandoned because it tends to result in starting point bias, an effect such that the final WTP amount at the end of the bidding game is systematically related to the initial bid value. Another disadvantage of the approach is that repeated questioning may annoy or tire respondents, causing them to say "yes" or "no" to a stated amount in hopes of terminating the interview.

This paper studies the ability- and willingness- to-pay of informal sector workers who have stopped paying the social health insurance, the Jaminan Kesehatan Nasional (JKN), premium for six months or more in Indonesia. The study uses a mixed methods approach. The authors exploit the responses of 1,709 individuals in 2016 to obtain the willingness-to-pay. Further, they use qualitative interviews to 53 of the respondents to explore in depth which factors explain ability- and willingness-to-pay.

The subject is interesting, and the paper is well written. I also believe the quantitative exercise has been executed correctly. The paper has the strength of combining different datasets and considering the regional differences in ATP, etc. Also, it provides a rich discussion of policy solutions to address the excess of uninsured individuals in Indonesia.
Nevertheless, I have some important caveats that would be addressed for this paper to be publishable at a journal such as PLOS One.

Major:

1. The title does not reflect well the contents of the paper. The study relies on informal sector workers that used to be insured and have failed to pay their premia for 6 months or more. There are 39 million informal sector workers that have never been enrolled and thus are not represented in the sample. The selected sample has characteristics that made them different than those that never have been enrolled. They had decided to pay in the past for whatever reason, while those that never enrolled did not. Therefore, I would recommend changing the title to echo the real content. Also, the authors should argue better why the selection is valid and why - even though the results are necessarily biased, they are useful.

**We agree with the reviewer and have adapted the title. The title has been changed to represent the main research objective and specify the study focuses on enrollees and not informal sector workers in general.**

2. The sample is now a bit dated: Being in 2021, to use a survey of 2016 may be a bit of a stretch. One solution would be to provide some statistics to show that the GDP per capita, inflation, etc. has not changed excessively since 2016 (ignoring 2020 as it is a year of complete disruption due to COVID).

**We have added a paragraph in the Discussion on the study’s limitations and mentioned the small changes in real GDP per capita since the survey’s implementation give us little reason to believe the interpretation of the results would change substantially.**

3. The method they use to estimate the WTP for health insurance should be better defended. In general, ‘this approach has been virtually abandoned because it tends to result in starting point bias, an effect such that the final WTP amount at the end of the bidding game is systematically related to the initial bid value.’ (Applications of the Contingent Valuation Method in Developing Countries: A Survey, by Anna Alberini, Joseph Cooper, Food and Agriculture Organization of the United Nations, Food & Agriculture Org., 2000 - Business & Economics). It can be argued that this is not a problem in this context, given that there is a point of reference (the premium), but then, the authors need to defend their stance.

**Thank you for pointing this out, and for your constructive suggestions. In the above-mentioned paragraph on the study’s limitations, we have also explained the grounding of the bidding game in a real-world value (i.e., the premium) which makes the interpretation of accepted bid value less spurious.**

4. What dictate the choice of explanatory variables in Table 7. The authors collected much more information from the individuals. The literature has shown that there are more explanatory variables that may influence health insurance decisions (gender of the head of household, number of children in the household, number of elderly, number of health shocks in the last year, etc.). A discussion on the choice of variables included in a regression is needed.

**We have tried to make this clearer and provide some information on the choice of variables. A sentence has been added to the Data Entry and Analysis sub-section which explains how the regressions were built, based on hypothesized associations between personal characteristics and WTP, whereupon the model was refined by removal of non-significant predictors.**

Minor:

1. The subsection called ATP and WTP could be written better to improve its readability. At the moment, the choice of style and formatting make it somewhat cumbersome.

**Many thanks for this comment. A few sentences in these subsection have been re-written to improve clarity.**

2. The references are outdated. There are a number of articles on WTP for health insurance of informal sector workers in low- and middle- income countries published since 2017.

**As mentioned in our response to Reviewer One’s comment, we have added several recent articles to the Introduction.**
